# Ultra-Low Ultraviolet Photon Detection of Diamondene Van der Waals Heterostructure by Interfacial Bonding

**DOI:** 10.34133/research.0806

**Published:** 2025-08-04

**Authors:** Jiapeng Zhen, Silin Guo, Ying Yue, Shuai Huang, Danping Zhang, Kai Shen, Kehong Lv, Qiushi Huang, Jing Qiu, Guanjun Liu

**Affiliations:** ^1^ College of Intelligence Science and Technology, National University of Defense Technology, Changsha, Hunan 410073, People’s Republic of China.; ^2^ National Key Laboratory of Equipment State Sensing and Smart Support, National University of Defense Technology, Changsha, Hunan 410073, People’s Republic of China.; ^3^ Beijing Computational Science Research Center, Beijing 100093, People’s Republic of China.

## Abstract

With the increasing demand for high sensitivity, low interference, and micro-size of deep ultraviolet spectral information in the field of photoelectric detection, low-dimensional diamond-based ultraviolet photoelectric detection has attracted great interest. However, although the diamond-based material has a high mobility, its lack of free electrons seriously hinders its conductivity and detection ability. Therefore, improving the free electron level of diamond-based materials has become the primary goal for achieving high-performance applications. Here, we demonstrate a diamondene photodetector formed by van der Waals heterostructure bonding, which has a high level of free electrons and is more advantageous for ultraviolet light. The diamondene photodetector realized by this method has a high responsivity of 2.04 A/W and D* higher than 10^12^ Jones, and the external quantum efficiency is as high as $1.1\times 10^{3}\%$. In the detection of imaging applications, ultraviolet imaging with very low photon number is realized, and clear imaging can be achieved only under the light intensity with a photon number of 28. It is of great substantial for the development and theoretical research of ultra-high sensitivity ultraviolet photodetectors in the future.

## Introduction

Ultraviolet (UV) photoelectric detection has the advantages of not easy to be disturbed and strong concealment. It is a key component of modern photoelectric technology [1]. It has unique advantages in imaging, aerospace, medical, communication, and other fields, which can make up for the shortcomings of other band detection [2]. Due to the increasing demand for weak optical signal sensing and strong anti-interference ability in modern equipment applications, the research on UV photoelectric detection has attracted more and more interest. At present, due to the limitation of material band gap, most of the photodetectors are concentrated in the visible-infrared band, but there are few studies in the UV band, especially in the 200- to 280-nm band [3]. High-performance and low-cost UV photodetectors are difficult to achieve [4]. At present, the frontier exploration of solar-blind UV photodetectors is mainly focused on GaN, Ga_2_O_3_, InGaAs [5,6], and other materials [1,7]. However, the response band range of these materials is easily interfered by other band signals. In addition, the complex defects of Ga_2_O_3_ itself greatly limit its practical application [8,9].

As a new generation of carbon-based semiconductor materials, diamond has the advantages of high carrier mobility (>2,000 cm^2^ V^−1^ S^−1^), wide band gap, and specific light response band range and has outstanding development prospects in the field of deep UV detection. Its photodetector also has outstanding advantages such as low dark current and high signal-to-noise ratio [10]. However, due to the low free electron ability of the diamond material itself, its conductivity is low, which seriously limits its application in the field of photoelectric detection [11,12]. At the same time, the physical mechanism of its photoelectric detection ability also lacks systematic and in-depth study [13]. High-performance exploration of diamond-based material hydrogenation [13] and nitrogen–boron doping [14] have the potential to improve their electrical properties. The understanding of the physical mechanism of its high-performance photoelectric detection needs to be further strengthened [15]. Therefore, it is a great challenge to realize the highly sensitive detection of diamond-based photoelectric detection. Therefore, the development of diamond-based photodetectors to improve their detection and imaging sensitivity [16,17], especially the light responsivity and imaging performance under weak light perception [18], is of great significance for the future application of diamond-based devices.

To solve the above challenges, we use the van der Waals (vdW) heterojunction bonding method to achieve a significant improvement in the free electron level of the low-dimensional diamondene material. The optical response band is pure, only in the 220- to 236-nm band. The responsivity is as high as 2.04 A/W at 230 nm. Through research, it is found that the device D* is higher than 10^12^ Jones, and the quantum efficiency is as high as $1.1\times 10^{3}\%$. At the same time, in the application of UV photoelectric imaging, the device can achieve detection imaging under extremely low photon conditions. When the photon number is 28, the imaging perception is still clear. Therefore, this progress opens up opportunities for the design and performance upgrade applications of high-sensitivity photodetectors in the future.

## Results

**Fig. 1.**
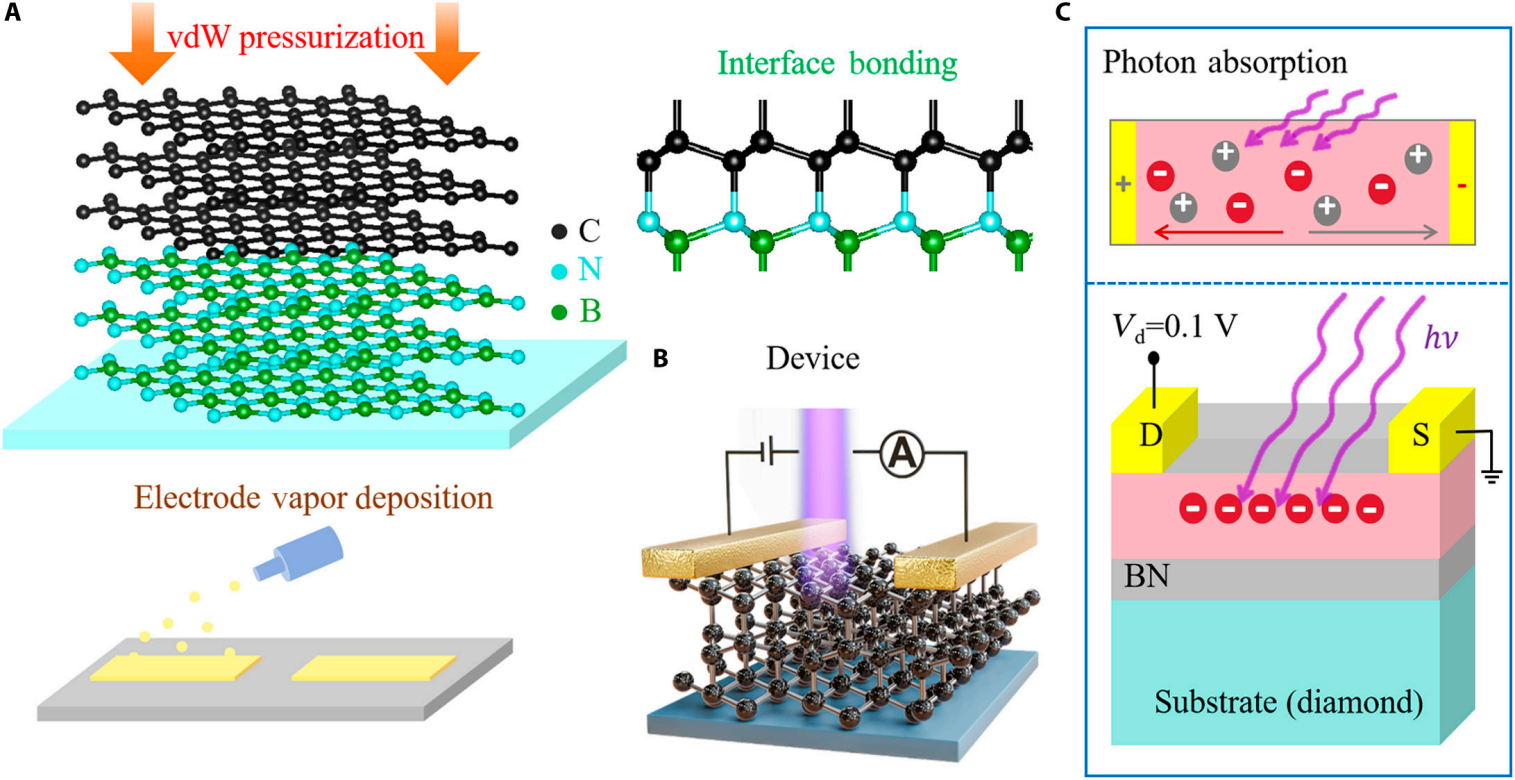
Principle and mechanism of diamond-based UV device. (A) Fabrication of diamond-based vdW heterostructure devices. (B) Interface structure and device. (C) Photon absorption and charge transport principle diagram of photodetector.

We used an interesting method to fabricate the device, which can effectively improve the carrier level of the device. The fabrication method of the device is shown in Fig. 1A. Firstly, the vdW heterostructure was constructed on the diamond substrate by using few-layer graphene and hexagonal boron nitride (hBN). The device electrode was etched by evaporation method, and then the photodetector with interface bonding structure was prepared by high-pressure irreversible bonding [14]. In terms of modulated electronic and optoelectronic properties, diamond-based materials have outstanding advantages in the field of deep UV photoelectric detection due to their wide band gap and higher carrier level than Ga_2_O_3_ [19,20]. Using the technology previously studied by our team, a diamond-based transistor photodetector device was prepared, and the basic schematic is shown in Fig. 1B [13]. Because the diamond substrate has good light transmission characteristics, it provides good support for photoelectric detection. Next, we further analyze its UV electron characteristics. When light with sufficient energy is irradiated on the material, the material will change its conductivity and then form pairs of holes and electrons [21]. The principle diagram of carrier photoelectric generation under light source illumination is shown in Fig. 1C. Usually, only photons with energy higher than the band gap of the material will be absorbed, and a pair of electrons and holes will be generated. These electrons and holes will increase the carrier level in the material and further improve their conductivity [1]. Therefore, when photons with energy greater than the band gap are incident, the channel material produces electrons and holes due to the photoelectric effect and then is separated by the applied built-in electric field. Subsequently, under the action of a bias electric field, electrons and holes move in the opposite direction that is easy to recombine, and the potential in the absorption layer increases, further increasing the electron density in the transport layer, resulting in positive light response characteristics [22].

**Fig. 2.**
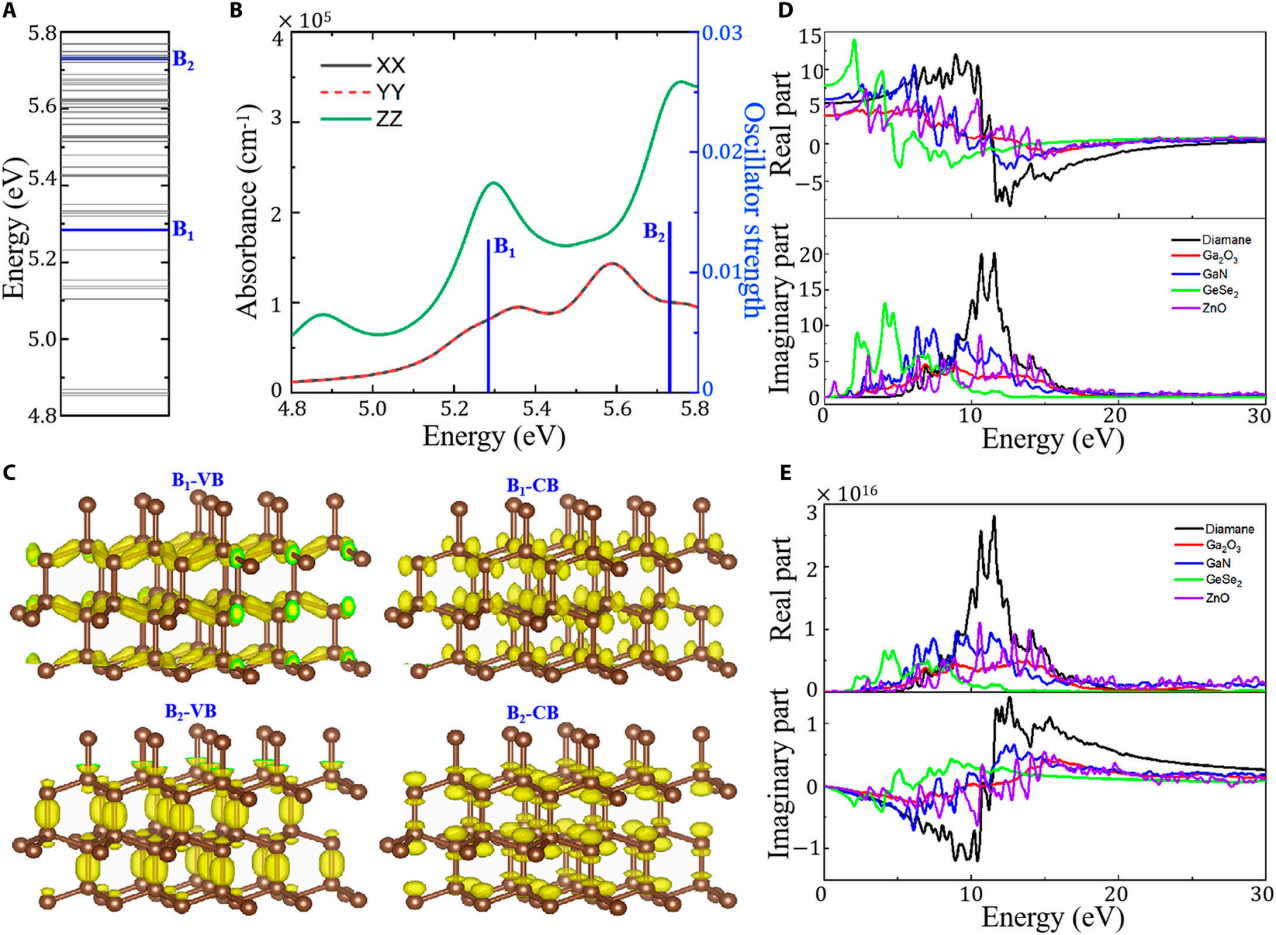
First-principles calculation of photoelectric correlation properties. (A) BSE exciton energy levels of diamond-based material. Dark and bright excitons are indicated by gray and blue, respectively. (B) BSE optical absorption spectrum of diamond-based material. The normalized oscillator strengths of the bright excitons are also presented. (C) Wave functions of the absorption energy bands forming bright excitons. The comparison of optical properties between diamane, Ga_2_O_3_, GaN, GeSe_2_, and ZnO (D) dielectric functions and (E) optical conductivity.

To further explore the electron and phonon dynamics of diamane structure under UV light excitation, the theoretical calculation of diamond-based structure model was performed using the GW-BSE (Bethe–Salpeter equation) method with density functional theory (DFT) (see Materials and Methods). We present the BSE exciton energy levels and optical absorption spectrum of the diamond-based material in Fig. 2. Here, excitons with an oscillator strength below 0.01 are considered dark excitons, represented in gray in Fig. 2A, while bright excitons are shown in blue. The 2 lowest bright excitons have excitation energies of 5.28 eV (B_1_) and 5.73 eV (B_2_), with large oscillator strengths, resulting in strong optical absorption as seen in Fig. 2B. It can be seen from the first 2 light absorption peaks that they correspond to wavelengths of 220 and 246 nm, which are in agreement with the experimental results. The wave functions corresponding to the absorption energy bands of the 2 bright excitons (B_1_ and B_2_) are illustrated in Fig. 2C. The intensity of the absorption peaks is governed by the transition matrix elements between these band wave functions, as dictated by Fermi’s golden rule. When subjected to an external electric field (e.g., solar-blind UV irradiation at 200 to 280 nm), electrons in the diamane lattice are photoexcited from the ground state to the excited state, leading to charge transfer between adjacent carbon orbitals. This electron transfer process modifies the local Coulomb potential, which subsequently perturbs the lattice vibrational modes (phonon dynamics) through electron–phonon coupling. Such synergistic interactions between charge carriers and phonons suppress nonradiative recombination losses, thereby enhancing the intensity of photocurrent generation. These findings establish a theoretical foundation for diamond-based photoelectric imaging devices, particularly in solar-blind UV detection, where low dark current and high signal-to-noise ratio are critical. Further performance improvements could be anticipated by engineering diamane nanostructures or introducing dopants to tailor the exciton–phonon coupling strength.

To elucidate the exceptional photodetection merits of diamane, we computed its complex dielectric function ε and derived critical optical parameters (such as optical conductivity σ, reflectivity *n*, extinction coefficient *k*, and absorption coefficient α) to systematically characterize its optoelectronic properties. A comparative analysis with β-Ga₂O₃, GaN, GeSe_2_, and ZnO further validated diamane’s superior photoresponse performance (Fig. 2D and E). Additionally, the reflectivity 𝑛 and the extinction coefficient 𝑘 are shown in Fig. S2. Diamane exhibits significantly stronger optical dispersion than β-Ga₂O₃ in the 5- to 15-eV spectral range, with its optical conductivity, reflectivity, and extinction coefficient demonstrating a steeper photon energy-dependent gradient and higher peak value. This confirms its superior sensitivity for UV photodetection applications. The photogenerated current density under illumination is governed by the formula J=q·G·η·μE, where *q* is the elementary charge, *G* represents the photogenerated carrier generation rate (dependent on light absorption coefficient α and light intensity I), η denotes the carrier separation efficiency, μ is the carrier mobility, and *E* is the applied electric field. Among the critical factors influencing photocurrent-carrier generation rate, separation efficiency, and mobility, diamane demonstrates significant advantages over other materials. Diamane’s far superior carrier mobility enables faster drift velocities of photogenerated carriers under an external field, directly enhancing current generation. In addition, unlike these compounds, diamane is a single-element material consisting only of carbon, which facilitates the preservation of its pure phase during synthesis. Furthermore, its high defect formation energy inhibits the formation of structural defects. In contrast, Ga₂O₃ and other oxides suffer from rapid carrier loss due to oxygen vacancy-induced defects and accelerated recombination at elevated temperatures [9]. Furthermore, diamane exhibits a distinct light absorption advantage: While weakly absorbing visible light, it displays a sharp absorption peak in the UV range with coefficients exceeding $10^{6}\;cm^{-1}$, far surpassing Ga₂O₃, GaN, GeSe_2_, and ZnO (Fig. S2). This strong UV absorption ensures a higher density of photogenerated carriers per unit volume in diamane, leading to a significantly enhanced baseline photocurrent under identical illumination conditions. These combined properties position diamane as a superior candidate for high-efficiency optoelectronic applications.

**Fig. 3.**
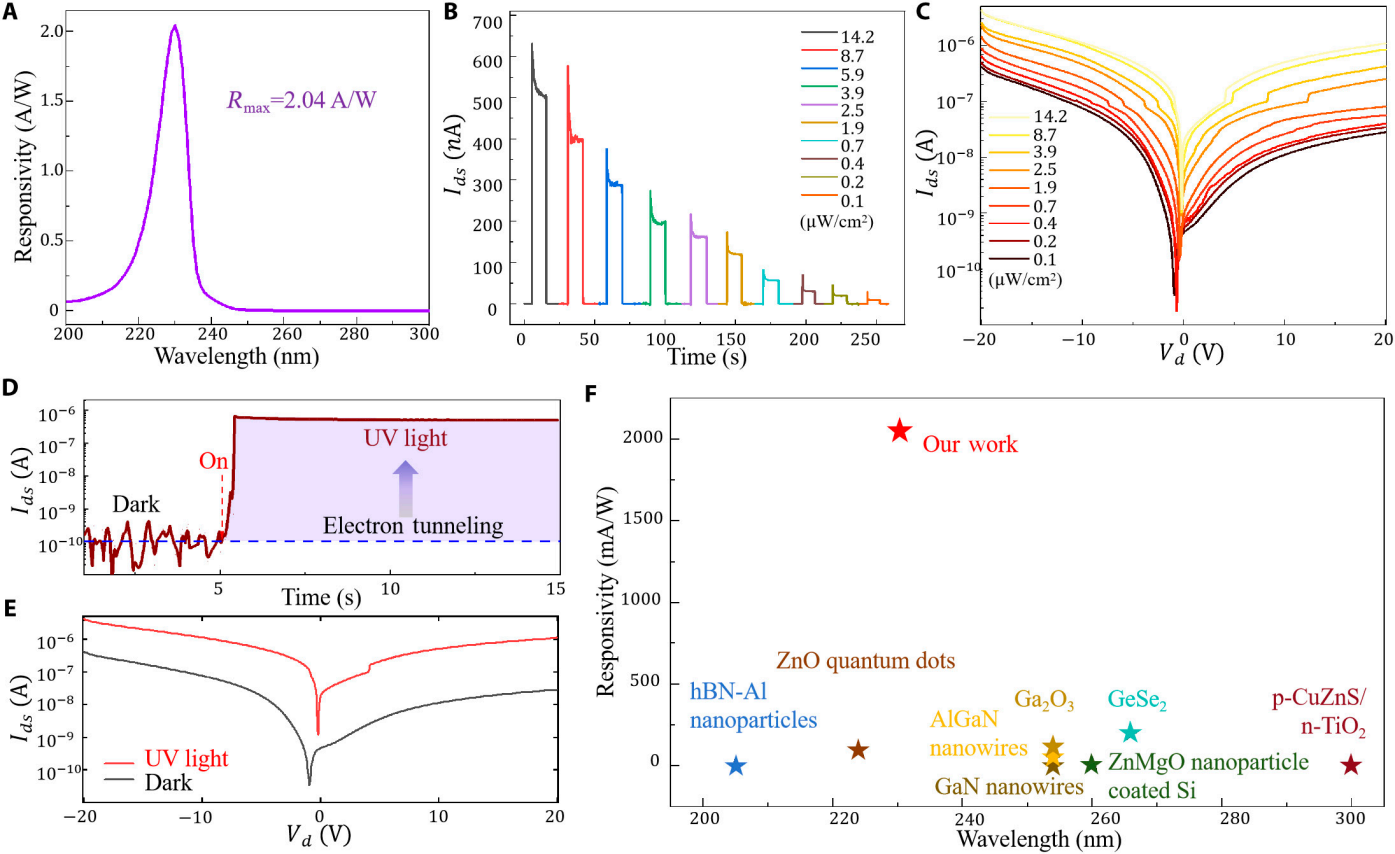
The light response characteristics of the diamond-based photodetector. (A) Light response results. (B) current–time (*I*–*T*) response under different light intensities. (C) *I*–*V* response under different light intensities. (D) Optical response of the device under 230-nm UV light irradiation. (E) Dark current and photocurrent comparison. (F) Comparison of photoelectric response performance of different materials in 200 to 300 nm.

To evaluate the photoelectric performance of diamond-based photodetectors, 2 important parameters [spectral responsivity (R) and external quantum efficiency (EQE)] are mainly studied. The calculation equation can be expressed as $R_{\lambda }=\frac{I_{\mathrm{ph}}-I_{d}}{P_{\lambda }S}$, $\mathrm{EQE}=\frac{\mathrm{hc}}{\mathrm{e\lambda}}R_{\lambda }$, where $I_{\mathrm{ph}}$ and $I_{d}$ represent the photo and dark current, respectively, $P_{\lambda }$ is the intensity density of the incident light with the wavelength of incident light, S is the effective area, and h, c, and e represent Planck constant, light speed, and electron charge, respectively. Figure 3A shows that the diamond-based device has a good optical response in the 220- to 246-nm band. When the device is biased at $V_{\mathrm{ds}}=20\;V$, the wavelength with the strongest optical response is 230 nm, and the corresponding maximum responsivity is 2.04 A/W, which is better than the photoelectric performance of Ga_2_O_3_ in this band (76.2 mA/W) [5], and also far superior to the traditional diamond photoelectric detection performance (45 mA/W) [12]. Meanwhile, the EQE of the device is measured as high as $1.1\times 10^{3}\%$. Furthermore, the detectivity ($D^{*}$) can be estimated as $D^{*}=\frac{R_{\lambda }}{{\left(2eI_{d}/S\right)}^{1/2}}$. So the calculation value reaches up to $1.2\times 10^{12}$ Jones at 230 nm, and the UV/visible rejection ratio reaches up to $8.1\times 10^{3}$. Figure 3B and Fig. S3 show the current–voltage (*I*–*V* ) characteristics of diamond-based photodetectors in the dark and under different light intensities at 230 nm. As the light intensity increases from 0.1 to 14.2 μW/cm², both zero-bias and negative-bias currents increase rapidly, which is attributed to the enhanced photocarrier generation, indicating a strong UV response. Additionally, considering the dependence of diamond-based photodetectors on external conditions, the effect of external bias on device performance was also tested. Figure 3C shows the highly repeatable switching behavior of the detector at a bias range of −20 to 20 V with a switching interval of 25 s. The on/off current ratio of the transistor is about 10^6^. The results show that the diamond-based photocurrent increases with the applied voltage, indicating that an increase in applied voltage effectively accelerates the separation of photogenerated carriers [23], and the photoelectric detection performance can be optimized by modulating the bias voltage. Meanwhile, under the light induction of 230-nm wavelength, the device has a good photocurrent effect in the voltage range of −20 to 20 V, and the difference between photocurrent and dark current is obvious (Fig. 3D and E). When the voltage increases from 0 to 20 V, the photocurrent of the detector increases rapidly to 1,000 nA, where the photocurrent is about 2,000 times of the dark current. The device has a fast response with a short rise and decay time of about 62 μs, as shown in Fig. S4. It shows that the device has good optical response performance under voltage bias, which provides strong support for subsequent imaging applications. Figure 3F and Table S1 clearly show the detection sensitivity level of the device studied in this paper in the range of 200 to 300 nm. The current diamond-based UV detection sensitivity is at a leading level, which is much higher than that of mainstream materials such as Ga_2_O_3_ and GaN [1].

**Fig. 4.**
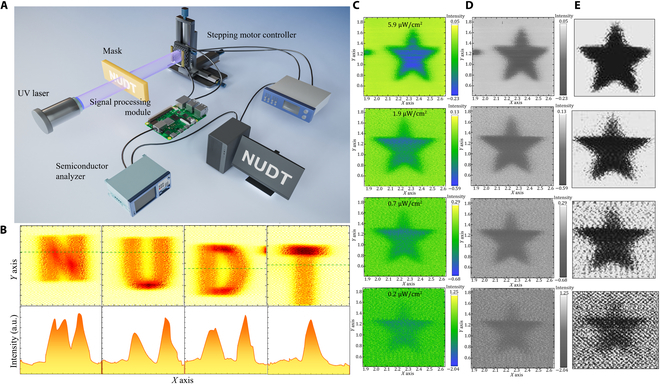
Diamond-based UV photoelectric imaging results under different light intensities. (A) Schematic diagram of the imaging detection system. (B) Photoelectric imaging results of the “NUDT” and the normalized intensity of the photocurrent corresponding to the dotted line. Image processing of “Pentagram” imaging under different light intensities. (C) Initial imaging result. (D) Gray image. (E) Linear noise reduction processing result.

Due to the high photosensitivity of diamond-based photodetectors in the “solar-blind” UV band, they hold great potential for image sensing applications. Therefore, this paper constructs an imaging system with diamond-based photodetectors as sensing pixels (Fig. 4A) to achieve high-sensitivity image detection. The experiment utilized a 230-nm light source, a movable 3-dimensional displacement platform, and a Keithley 2450 source meter. The light source was positioned in front of an image mask, and the light beam passed through the hollow pattern to irradiate the diamond-based photodetector. The motion of the diamond-based photodetector was controlled by the *X*-*Y* plane mobile platform, enabling multi-physical field combination imaging. A computer connected to the semiconductor analyzer recorded the spatial resolution optical response of the diamond-based device in real time.

Through scanning imaging, under a 230-nm UV light signal of 3.9 $\mathrm{\mu W}/{\mathrm{cm}}^{²}$, a high-resolution “NUDT” deep UV image was obtained, as shown in Fig. 4B. It is evident that the image pattern of the diamond-based UV photodetector under weak UV light intensity is clear, with well-defined edge details. To further evaluate the consistency between the UV imaging and the target, the straight line and cross-section of the continuous current values in Fig. 4B were selected. The current response transformation is evident and aligns with the clear boundary of the UV image, indicating that diamond-based materials can serve as sensing pixels to obtain high-fidelity images under weak UV signals. These impressive results clearly demonstrate the imaging application prospects of diamond-based deep UV phototransistors.

To further verify the imaging detection capability of diamond-based photodetectors in weak solar-blind UV light signals, this paper compares the pentagram target imaging across UV light intensities ranging from 0.2 to 5.9 μW/cm², as shown in Fig. 4C. It can be observed that the imaging results of the target under different light intensity conditions exhibit clear patterns and distinct edge detail contrasts. When the light intensity is reduced to very low levels (0.2 μW/cm²), the signal-to-noise ratio between the target and the surrounding environment is poor, but the specific contours of the pentagram remain identifiable. This demonstrates that despite the attenuation of UV light power, the diamond-based photodetector still possesses excellent single-point imaging capability under weak light conditions.

To better discuss the detection sensitivity of the photodetector, we calculated the optical power intensity to the number of photons. The expression of single-photon energy is $E_{1}=\frac{\mathrm{hc}}{\lambda }$, Therefore, we estimate that the number of photons obtained by each sampling at 0.2 μW/cm² light intensity is about 28 at 230 nm, which confirmed that the detector can achieve clear detection imaging at extremely low photon number. To apply postprocessing to the experimental imaging and verify its feasibility, this paper further employs a linear processing method for image enhancement. So we performed image optimization processing on pentagram imaging under different UV light intensities in sequence. During the image processing, the image features were first gray-scaled (Fig. 4D), and the target area was selected for uniform scale amplification. By adjusting the parameters, the final settings of the image were linearly transformed to obtain the final processed target image, as shown in Fig. 4E. The results indicate that the photoelectric scanning imaging results are capable of identifying the appearance of the “pentagram” even at ultra-low UV photon of 28. The difference in imaging under various light intensities lies in the varying noise intensity in the nontarget areas, which can be effectively denoised during subsequent image processing. Through the comparison of the responsivity of different materials (Fig. 3F and Table S1), it is found that the detection sensitivity of the device prepared in this paper at 230 nm is more than 20 times higher than that of the device prepared by other materials (ZnO quantum dots). It can be estimated that in the same scenario, the photon limit detected by other devices is higher than 1,000, which is far from the detection level of diamane-based detectors. Therefore, combined with the backend image recognition processing, the diamond-based photodetector presented in this paper can still clearly detect the target imaging under extremely weak solar-blind UV light signals, thereby confirming the high stability and photosensitivity of the solar-blind UV imaging system.

## Conclusion

In this study, we explored the mechanism and imaging applications of diamond-based UV photodetectors. We used the diamond-based vdW heterostructure bonding method to significantly improve the free electron level of the device and enhance its photoelectric detection capability. The optical properties of diamond and Ga_2_O_3_ were compared by first-principles calculations, which confirmed the outstanding potential of diamond in the field of deep UV photoelectric detection. Through the performance test of the prepared diamondene photodetector, it is found that the detector only has an optical response in the 220- to 236-nm band, the highest responsivity is 2.04 A/W (230 nm), the EQE is as high as $1.1\times 10^{3}\%$, and the D* is higher than 10^12^ Jones. Finally, we realized UV photoelectric imaging under extremely weak light intensity. Only under the excitation of 28 photons, the imaging pattern is clear and distinguishable, which provides strong support for the design and application of UV ultra-sensitive photodetectors in the future.

## Materials and Methods

### DFT calculations

DFT calculations were performed using the Vienna ab initio simulation package (VASP) [24], and the core-valence interaction was described by the projector-augmented wave (PAW) method [25]. The generalized gradient approximation of Perdew–Burke–Ernzerhof (GGA-PBE) exchange-correlation functional [26] was adopted. The excitonic and optical properties were obtained by solving the BSE [27,28] based on the *G*_0_*W*_0_ calculations.

### Device fabrication and characterization

We fabricated a diamond-based photodetector by the previous high-pressure synthesis method [14]. Electronic and optoelectronic performance tests were performed using an Agilent 4155C semiconductor analyzer and a standard electrical probe station under 230-nm UV light sources and a sampling frequency of 500 Hz.

See the Supplementary Materials for more details.

## Data Availability

All study data are included in the main text.
