# Supplementary material for: Ultra-Low Ultraviolet Photon Detection of Diamondene Van der Waals Heterostructure by Interfacial Bonding
Source: Research (Wash D C). 2025 Aug 4;8:0806. doi: 10.34133/research.0806 (PMC12320779; doi:10.34133/research.0806)
Supplement: Supplementary 1 — Supplementary Materials and Methods Figs. S1 to S4 Table S1 [file research.0806.f1.docx]

**Ultra-low ultraviolet photon detection of diamond-based vdW heterostructures by enhanced interfacial bonding**

Jiapeng Zhen^1,2^,, Silin Guo^1^, Ying Yue^3,*^, Shuai Huang^1,2^, Danping Zhang^1,2^, Kai Shen^1,2^, Kehong Lv^1,2^, Qiushi Huang^3,*^, Jing Qiu^1,2^, Guanjun Liu^1,2,*^

^1^College of Intelligence Science and Technology, National University of Defense Technology, Changsha, Hunan 410073, People’s Republic of China

^2^National Key Laboratory of Equipment State Sensing and Smart Support, National University of Defense Technology, Changsha, Hunan 410073, People’s Republic of China

^3^Beijing Computational Science Research Center, Beijing 100093, People’s Republic of China

*Email: [yueying@csrc.ac.cn](mailto:yueying@csrc.ac.cn), [qiushihuang@csrc.ac.cn](mailto:qiushihuang@csrc.ac.cn), [liu342@nudt.edu.cn](mailto:liu342@nudt.edu.cn)

1. **Materials and Methods**
   - - 1. **Device fabrication and characterization**

The irreversible phase change was used to prepare diamondene materials on diamond substrates to form diamondene-based photodetectors[1]. In this article, the irreversible bonding phase transition of graphene and hBN vdW heterostructures under high pressure is used to open the band gap of graphene to form diamondene. The basic characterization results of the materials of the device are shown in Fig. S1. The Raman spectrum has no G peak of graphene sp2 hybrid mode, and a stable band gap has been formed.

- - - 1. **Analysis of optical properties by first-principles calculation**

The real ($\varepsilon_{1}$) and imaginary ($\varepsilon_{2}$) components of the dielectric function were derived via self-consistent iterative computations. Therefore, the energy-dependent optical conductivity σ(E) is determined via the fundamental relation[2]: $\sigma\left( E \right)=\sigma_{1}\left( E \right)+i\sigma_{2}\left( E \right)=-i\frac{E}{4\pi\hbar}[\varepsilon\left( E \right)-1]$.

the reflectivity 𝑛 and the extinction coefficient 𝑘 are obtained from the relations[3] $\varepsilon_{1}=n^{2}-k^{2}$ and.

- - - 1. **Analysis of optical properties by first-principles calculation**

The wavefunctions were expanded using a plane-wave basis with a 300 eV kinetic energy cutoff. The thresholds for total energy convergence and structural relaxation are 10^-8^ eV and 10^-2^ eV/Å, respectively. Unless otherwise specified, a 15×15×6 Γ-centered Monkhorst-Pack k-point mesh[4] is used to sample the Brillouin zone. Quasiparticle energies are calculated using the single-shot GW (G_0_W_0_) approach[5] including 288 bands. The excitonic and optical properties were obtained by solving the Bethe-Salpeter equation (BSE)[6] based on the G_0_W_0_ calculations. Three valence bands and three conduction bands were included in the BSE optical transition calculations.

- - - 1. **Imaging processing method**

The basic principle of this method is to control the slope of the piecewise linear function and adjust the position of the inflection point through piecewise linear transformation, thus expanding or compressing the grayscale range of the image. This method is particularly suitable for cases where noise interference exists near the target or background region[7][8]. The piecewise linear function expression is as follows:

$$g(x,y)=\left\{\begin{matrix} \frac{c}{a}f(x,y)\begin{matrix} \begin{matrix} \begin{matrix} & \end{matrix} & & \end{matrix} & & 0<f(x,y)<a \end{matrix} \\ \frac{d-c}{b-a}\left[f(x,y)-a\right]+c\begin{matrix} \begin{matrix} & \end{matrix} & a\leq f(x,y)\leq b \end{matrix} \\ \frac{g_{\max}-d}{f_{\max}-b}\left[f(x,y)-b\right]+d\begin{matrix} & b<f(x,y)\leq f_{\max} \end{matrix} \end{matrix}\right.$$

Where, $a$、$b$、$c$、$d$are four adjustable parameters, $f(x,y)$and$g(x,y)$are the$X$axis and the$Y$axis pixel function respectively. By adjusting the adjustable parameters to transform the grayscale between images, the noise effect around the target can be reduced.

1. **Figures**


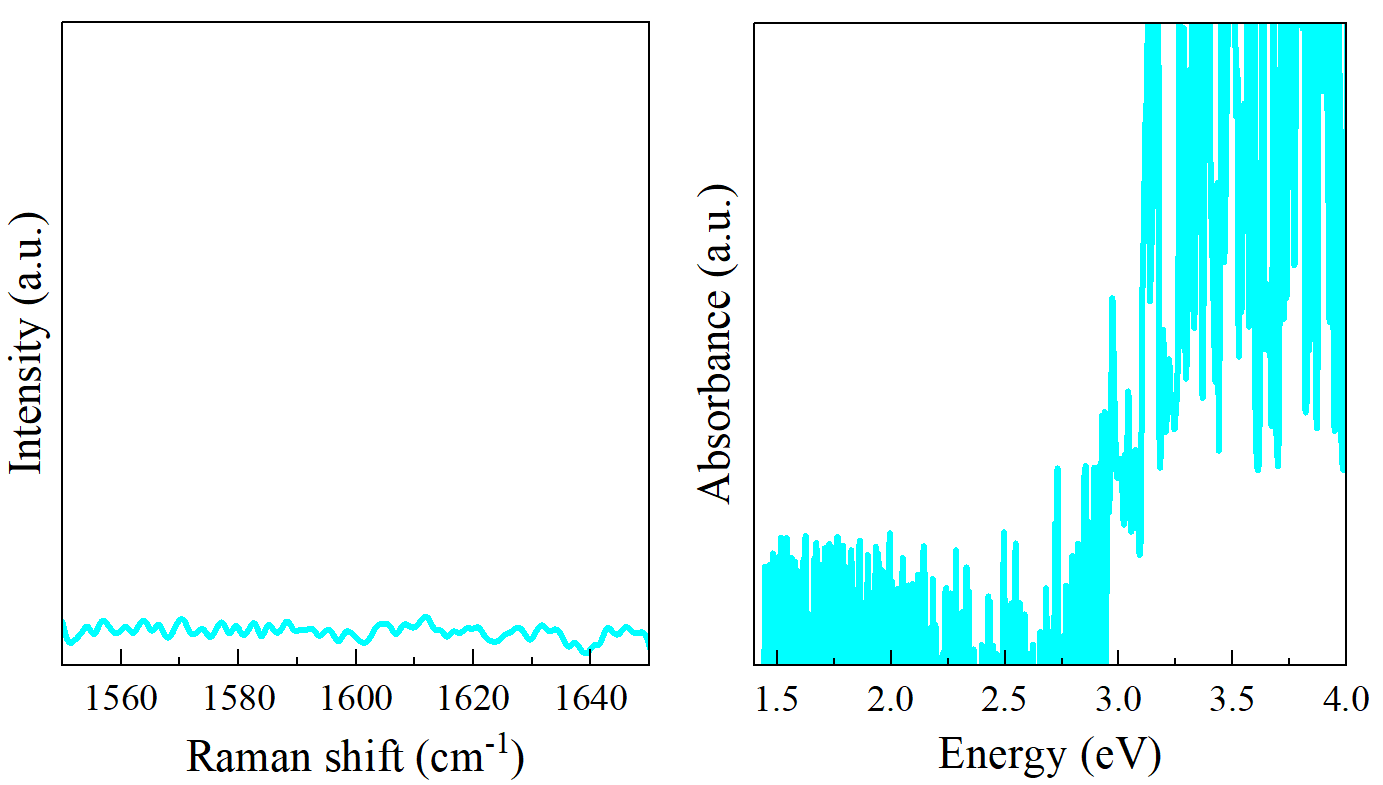


Fig. S1 Basic characterization of diamondene-based devices.


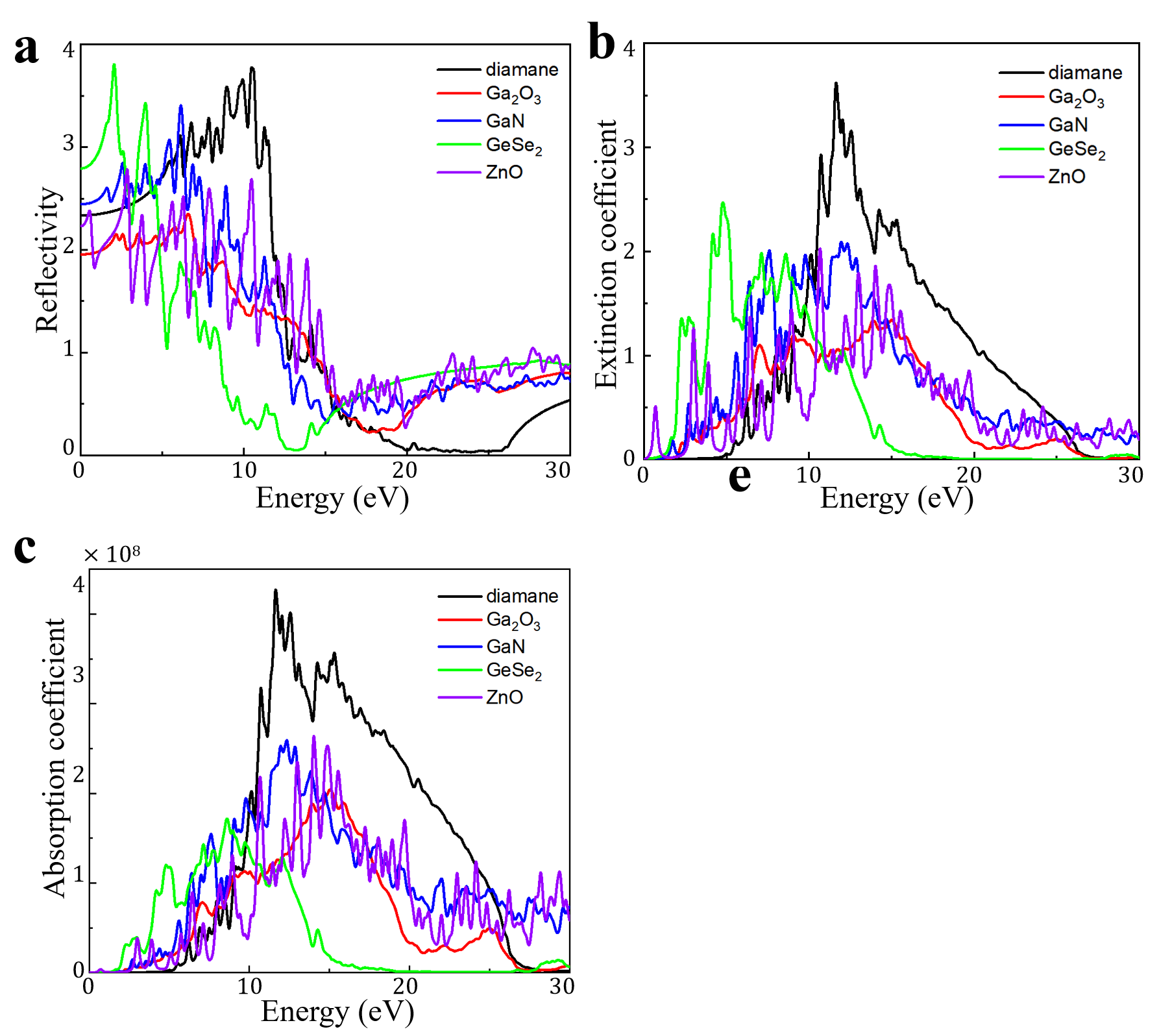


Fig. S2 The comparison of optical properties between diamane, Ga_2_O_3_, GaN, GeSe_2_ and ZnO of (a) reflectivity, (b) extinction coefficient and (c) absorption coefficient.


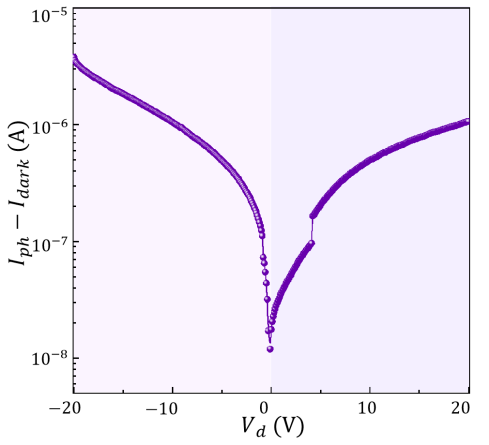


Fig. S3 The net photocurrent change of the device.


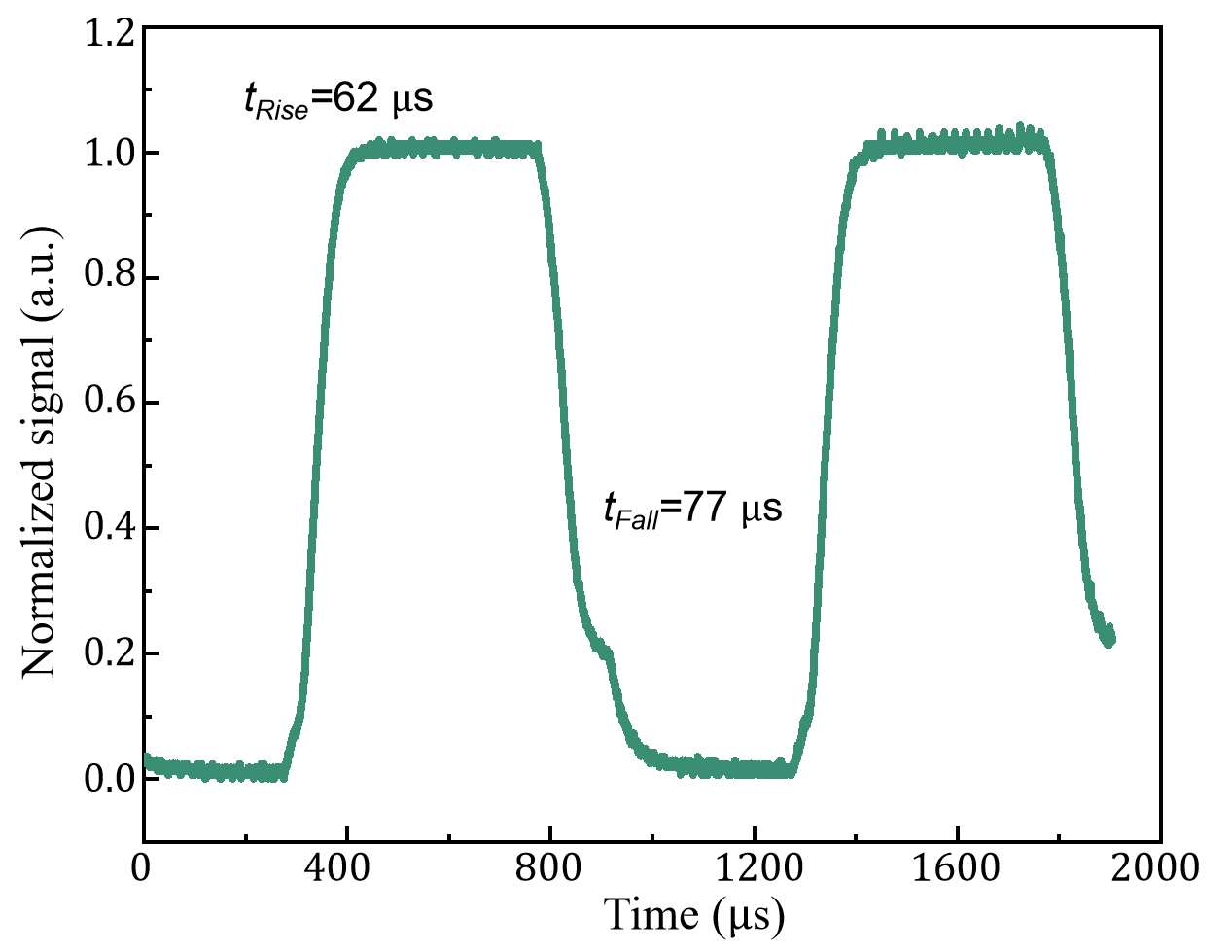


Fig. S4 The time-dependent photoresponse curve.

1. **Tables**

Table S1 Comparison of photoelectric response performance of different materials in 200-300 nm.

| Material | Response center wavelength (nm) | Responsitivity (mA/W) |
| --- | --- | --- |
| diamond | 230 | 2040 |
| ZnO quantum dots | 224 | 100 |
| n-GaN nanowires | 254 | 3.7 |
| AlGaN nanowires | 254 | 48 |
| p-CuZnS/n-TiO_2_ arrays | 300 | 2.54 |
| hBN-Al nanoparticles | 205 | 0.012 |
| Ga_2_O_3_ | 254 | 120 |
| ZnMgO nanoparticle coated Si | 260 | 7.5 |
| GeSe_2_ | 266 | 200 |

1. **Reference**
2. Zhen J, et al. Irreversible coherent matching bonding of van der Waals heterostructure lattice by pressure. *PNAS*. **121**(23), e2403726121 (2024).
3. Z. Luo, et al. First-principles study of electronic and optical properties of BiTiO3. *Acta Physica Sinica*, **64**, 147102 (2015).
4. H. Liu, et al. Study on characterization method of optical constants of germanium thin films from absorption to transparent region. *Materials Science in Semiconductor Processing*, **83**, 58-62 (2018).
5. H. J. Monkhorst et al. Special points for Brillouin-zone integrations. *Phys. Rev. B* **13**, 5188 (1976).
6. M. Shishkin et al. Implementation and performance of the frequency-dependent GW method within the PAW framework. *Phys. Rev. B* **74**, 035101 (2006).
7. M. Rohlfing et al. Electron-Hole Excitations in Semiconductors and Insulators. *Phys. Rev. Lett.* **81**, 2312 (1998).
8. A. M. Molaei et al.Fast Processing Approach for Near-Field Terahertz Imaging With Linear Sparse Periodic Array. *IEEE Sensors Journal* **22**(5): 4410-4424 (2022).
9. L. Fan et al. Brief review of image denoising techniques. *Vis. Comput. Ind. Biomed. Art* **2**, 7 (2019).
